# Biochemical characterization of Cdk2-Speedy/Ringo A2

**DOI:** 10.1186/1471-2091-6-19

**Published:** 2005-09-28

**Authors:** Aiyang Cheng, Shannon Gerry, Philipp Kaldis, Mark J Solomon

**Affiliations:** 1 Department of Molecular Biophysics and Biochemistry, Yale University School of Medicine, 333 Cedar Street, New Haven, CT 06520-8024, USA; 2 Mouse Cancer Genetics Program, National Cancer Institute, Frederick, MD 21702-1201, USA; 3 Department of Biological Sciences, University of Rhode Island, Kingston, RI 02881, USA

## Abstract

**Background:**

Normal cell cycle progression requires the precise activation and inactivation of cyclin-dependent protein kinases (CDKs), which consist of a CDK and a cyclin subunit. A novel cell cycle regulator called Speedy/Ringo shows no sequence similarity to cyclins, yet can directly bind to and activate CDKs. Speedy/Ringo proteins, which bind to and activate Cdc2 and Cdk2 *in vitro*, are required for the G2 to M transition during *Xenopus *oocyte maturation and for normal S-phase entry in cultured human cells.

**Results:**

We have characterized the substrate specificity and enzymatic activity of human Cdk2-Speedy/Ringo A2 in order to gain insights into the possible functions of this complex. In contrast to Cdk2-cyclin A, which has a well-defined consensus target site ((S/T)PX(K/R)) that strongly favors substrates containing a lysine at the +3 position of substrates, Cdk2-Speedy/Ringo A2 displayed a broad substrate specificity at this position. Consequently, Cdk2-Ringo/Speedy A2 phosphorylated optimal Cdk2 substrates such as histone H1 and a KSPRK peptide poorly, only ~0.08% as well as Cdk2-cyclin A, but non-canonical Cdk2 substrates such as a KSPRY peptide relatively well, with an efficiency of ~80% compared to Cdk2-cyclin A. Cdk2-Speedy/Ringo A2 also phosphorylated authentic Cdk2 substrates, such as Cdc25 proteins, which contain non-canonical CDK phosphorylation sites, nearly as well as Cdk2-cyclin A. Phosphopeptide mapping indicated that Cdk2-Speedy/Ringo A2 and Cdk2-cyclin A phosphorylate distinct subsets of sites on Cdc25 proteins. Thus, the low activity that Cdk2-Speedy/Ringo A2 displays when assayed on conventional Cdk2 substrates may significantly underestimate the potential physiological importance of Cdk2-Speedy/Ringo A2 in phosphorylating key subsets of Cdk2 substrates. Unlike Cdk2-cyclin A, whose activity depends strongly on activating phosphorylation of Cdk2 on Thr-160, neither the overall catalytic activity nor the substrate recognition by Cdk2-Speedy/Ringo A2 was significantly affected by this phosphorylation. Furthermore, Cdk2-Speedy/Ringo A2 was not a suitable substrate for metazoan CAK (which phosphorylates Cdk2 at Thr-160), supporting the notion that Speedy/Ringo A2 activates Cdk2 in a CAK-independent manner.

**Conclusion:**

There are major differences in substrate preferences between CDK-Speedy/Ringo A2 and Cdk2-cyclin complexes. These differences may accommodate the CAK-independent activation of Cdk2 by Speedy/Ringo A2 and they raise the possibility that CDK-Speedy/Ringo A2 complexes could phosphorylate and regulate a subset of non-canonical CDK substrates, such as Cdc25 protein phosphatases, to control cell cycle progression.

## Background

Eukaryotic cell cycle progression is under the control of cyclin-dependent kinases (CDKs). In higher eukaryotic cells, Cdc2, Cdk2, Cdk4, and Cdk6 control cell cycle progression. Their activities are regulated through a variety of mechanisms, including association with regulatory subunits (cyclins, inhibitors, and assembly factors), subcellular localization, transcriptional regulation, selective proteolysis, and reversible protein phosphorylation (reviewed in [1-7]).

Binding of a cyclin to a CDK is a crucial step in its activation and leads to its phosphorylation on multiple sites [5-8]. Activating phosphorylation within the activation segment (also called the T-loop) on a conserved threonine residue (Thr-160 in Cdk2) is required for CDK activities *in vitro *and *in vivo *[9-16] and is carried out by the Cdk-activating kinase (CAK), which is composed of Cdk7, cyclin H, and Mat1 in most eukaryotes. Inhibitory phosphorylations occur on sites equivalent to Thr-14 and Tyr-15 in human Cdc2 or Cdk2 and are carried out by Wee1-like protein kinases (Wee1 and Myt1), and removed by members of the Cdc25 phosphatase family (reviewed in [6,7]). In addition to activating CDKs, cyclins also contribute to their substrate specificities [17-20] and to their subcellular localizations [21]. A general consensus sequence for efficient phosphorylation by Cdc2 and Cdk2 is (K/R)(S/T)PX(K/R) [22-24], in which a basic residue at the +3 position three amino acids C-terminal to the phosphorylated Ser or Thr is particularly important.

In addition to their activation by cyclins, CDKs can be activated by a novel cell cycle regulator called Speedy or Ringo (Rapid inducer of G_2_/M progression in oocytes), despite the lack of any primary sequence homology between cyclins and Speedy/Ringo proteins [25-28]. Speedy/Ringo proteins were initially described in *Xenopus *based on their ability to promote the G2 to M transition during oocyte maturation [25,26]. *Xenopus *Speedy/Ringo bound to and activated Cdc2 and Cdk2 *in vitro *[26,27]. A human Speedy/Ringo homologue (Spy1) is essential for S-phase entry in cultured somatic cells in a Cdk2-dependent manner: overexpression of human Spy1 accelerated S-phase entry and cell proliferation, and its inhibition by RNAi caused a cell cycle delay at G1/S [28]. These biological functions of Speedy/Ringo proteins were dependent on CDK activity since kinase-inactive forms of Cdc2 and Cdk2 abolished the effects of Spy1 on cell cycle transitions [26,28]. Interestingly, unlike CDK-cyclin complexes whose phosphorylation on the activating site is essential for activity, Speedy/Ringo proteins can activate Cdc2 and Cdk2 *in vitro *in the absence of activating phosphorylation of the CDK [27]. Thus, *Xenopus *Speedy/Ringo could activate both wild type Cdk2 and Cdk2^T160A ^equally well and preincubation of Cdk2 with budding yeast CAK (Cak1p) had no effect on its kinase activity toward substrates such as histone H1 [27]. Cdk2-Speedy/Ringo complexes are also resistant to the inhibitory effects of p21^Waf1/Cip1 ^and Wee1 [27]. Paradoxically, although this ability of CDK-Speedy/Ringo complexes to bypass many forms of regulation imposed on CDK-cyclin complexes might suggest that CDK-Speedy/Ringo complexes would be highly active, only very low enzymatic activity toward conventional substrates is associated with CDK-Speedy/Ringo complexes isolated from cells (unpublished data).

The importance of Speedy/Ringo proteins for cell cycle progression combined with the low protein kinase activity of CDK-Speedy/Ringo complexes toward conventional substrates raised the possibility that there might be major biochemical differences between CDK-Speedy/Ringo complexes and CDK-cyclin complexes. These differences might explain how CDK-Speedy/Ringo complexes recognize their substrates and promote cell cycle progression. Major differences are to be expected since the activating phosphorylation that is dispensable for Cdk2-Speedy/Ringo activity plays a key role in substrate recognition by Cdk2-cyclin complexes [29,30]. Activating phosphorylation also enhances substrate binding by CDK-cyclin complexes [30]. The crystal structure of Cdk2-cyclin A with an optimal peptide substrate showed that the activating phosphate forms a hydrogen bond with the side-chain of the lysine at the +3 position of the substrate [31], providing insight into how the activating phosphate participates in substrate recognition. Although Cdk5 can be activated by p35/p25 (cyclin subunit of Cdk5) without activating phosphorylation, a glutamic acid side-chain in p35/p25 mimics the activating phosphate and interacts with a basic residue at the +3 position of the substrate [32]. It will be interesting to know how CDK-Speedy/Ringo complexes recognize substrates efficiently and how an apparently low level of CDK-Speedy/Ringo activity can have profound effects on cell cycle progression. A detailed biochemical understanding of CDK-Speedy/Ringo activity should help us to understand the biological functions of Speedy/Ringo proteins during cell cycle transitions.

In this study, we explored the biochemical properties of human Cdk2-Speedy/Ringo A2. We found that neither its overall catalytic activity nor its substrate recognition required the activating phosphorylation of Cdk2. In fact, Cdk2-Speedy/Ringo A2 was a poor substrate for phosphorylation by metazoan CAK. Cdk2-Speedy/Ringo A2 tolerated almost any amino acid residue at the +3 position of substrates, which is strikingly different from the rigid requirement of Cdk2-cyclin A and Cdk2-cyclin E for a basic residue (and, in particular, for a lysine) at the +3 position. Although Cdk2-Speedy/Ringo A2 phosphorylated canonical Cdk2-cyclin A substrates such as histone H1 and a KSPRK peptide quite poorly, it phosphorylated non-canonical CDK-cyclin substrates, including Cdc25 protein phosphatases, nearly as well as Cdk2-cyclin A. These observations raise the possibility that Cdk2-Speedy/Ringo could phosphorylate and regulate a subset of non-canonical CDK substrates, such as Cdc25 protein phosphatases.

## Results

### Characterization of purified proteins

**Figure 1:**
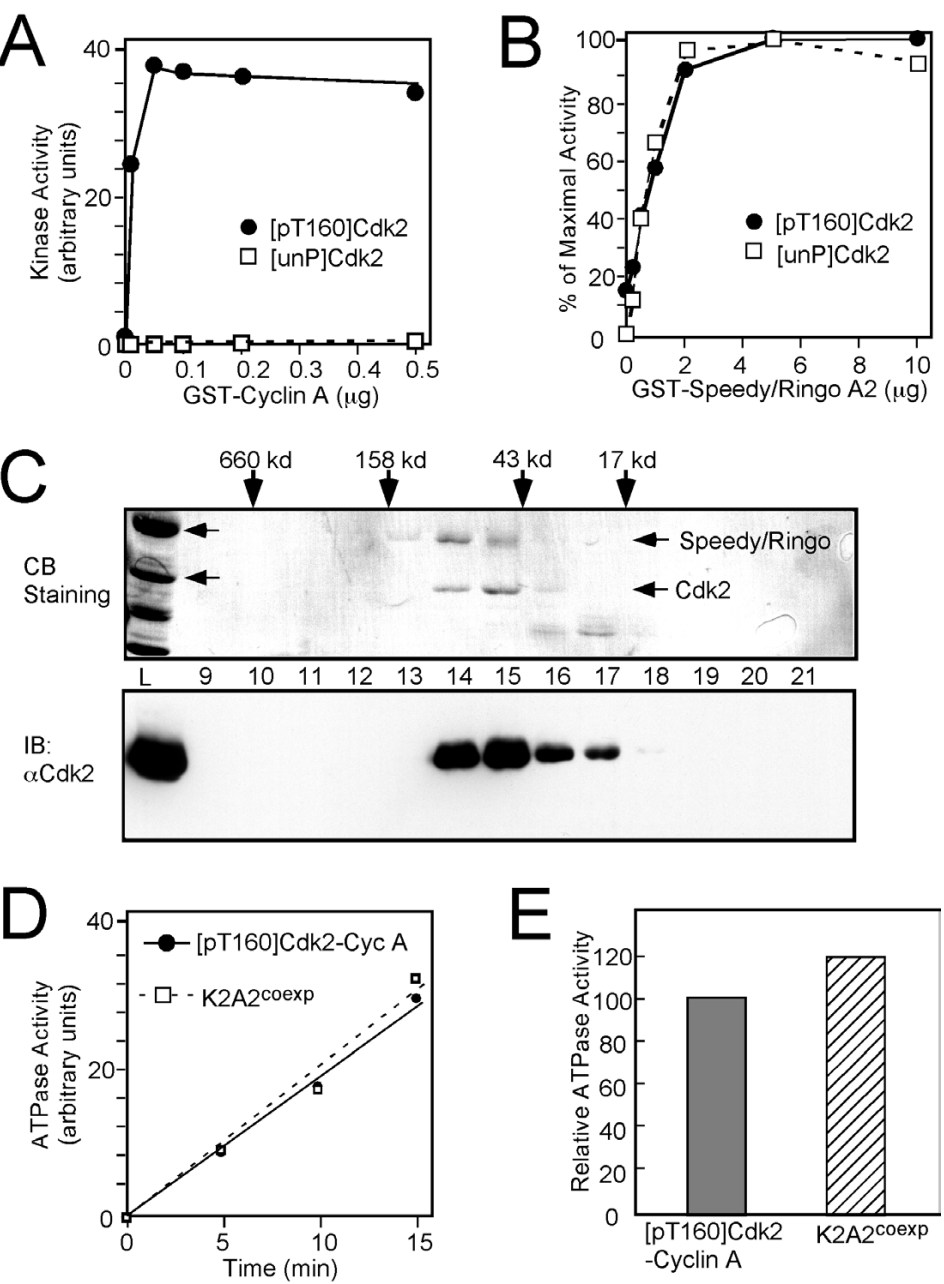
**Characterization of [unP]Cdk2, [pT160]Cdk2, Speedy/Ringo A2, and K2A2^coexp^**. (A) Activation of [unP]Cdk2 and [pT160]Cdk2 by cyclin A. 0.05 μg of [unP]Cdk2 or [pT160]Cdk2 was preincubated with the indicated amounts of cyclin A prior to determination of its histone H1 kinase activity. (B) Activation of [unP]Cdk2 and [pT160]Cdk2 by Speedy/Ringo A2. 0.5 μg of [unP]Cdk2 or [pT160]Cdk2 was preincubated with the indicated amounts of GST-Speedy/Ringo A2 prior to determination of its histone H1 kinase activity. (C) Gel filtration analysis of K2A2^coexp^. Cdk2 and Speedy/Ringo A2-His_6_ were coexpressed in *E. coli *and purified on a metal affinity column. The purified K2A2^coexp ^was loaded on a Superdex-200 column; one-ml fractions were collected. Proteins from 20 μl of the input (*lane L*) or from 40 μl of fractions 9–21 were resolved by SDS-PAGE, transferred to a PVDF membrane, and detected with a Cdk2-specific antibody (*lower panel*), or by staining with Coomassie Brilliant Blue R-250 (*upper panel*). (D) Time course of ATPase activity of [pT160]Cdk2-cyclin A and K2A2^coexp^. 16.7 μM of [pT160]Cdk2-cyclin A or K2A2^coexp ^was incubated with [γ^-32^P]ATP for the indicated times. Samples were chromatographed to resolve ^32^P_i _from [γ^-32^P]ATP. The rate of ATP hydrolysis was quantitated by phosphorimaging analysis. (E) The relative ATPase activities of [pT160]Cdk2-cyclin A and K2A2^coexp ^were calculated from the slopes in panel D. The ATPase activity of [pT160]Cdk2-cyclin A was set to 100%.

We initiated our work by purifying and characterizing the Cdk2, cyclin A, and Speedy/Ringo A2 proteins that would be used throughout these studies. Unphosphorylated GST-Cdk2 ([unP]Cdk2) was expressed in *E. coli *and purified via its GST tag. To produce Thr160-phosphorylated Cdk2 ([pT160]Cdk2), we used a previously described Cdk2-Cak1p co-expression system [31,33] that can produce essentially fully phosphorylated Cdk2 [31]. As previously reported [29,33], [pT160]Cdk2 by itself displays a low but detectable histone H1 kinase activity (Fig. 1A–B). Cyclin A stimulated the histone H1 kinase activity of [pT160]Cdk2 (solid circle) about 50-fold. The activation of [pT160]Cdk2 by cyclin A plateaued when the ratio of Cdk2 to cyclin A was about 1:1. In contrast to [pT160]Cdk2, the histone H1 kinase activity of [unP]Cdk2 (open squares) remained very low, even at high cyclin A concentrations, confirming the importance of the activating phosphorylation of Cdk2 on Thr-160 (Fig. 1A).

We next determined the histone H1 kinase activities of [unP]Cdk2 and [pT160]Cdk2 after incubation with GST-Speedy/Ringo A2, which was expressed and purified from *E. coli*. We have found that the previously reported mammalian Speedy/Ringo protein, Spy1, is expressed as two closely related proteins resulting from alternative splicing [34]. These two proteins, which we term Speedy/Ringo A1 and A2, differ only at their extreme C-termini and appear to be indistinguishable in all functional respects. Spy1 corresponds to Speedy/Ringo A1. As shown in Fig. 1B, GST-Speedy/Ringo A2 activated [unP]Cdk2 (open squares) and [pT160]Cdk2 (solid circles) equally well, indicating that Speedy/Ringo A2 can bind to and activate Cdk2 without regard to its activating phosphorylation. Cdk2 activity plateaued at a GST-Speedy/Ringo A2 to GST-Cdk2 ratio of at least 4:1. We suspect that an excess of GST-Speedy/Ringo A2 over GST-Cdk2 was required because much of the GST-Speedy/Ringo A2 protein aggregates, as we have observed by gel-filtration chromatography (data not shown), thereby reducing the effective concentration of active Speedy/Ringo A2.

As an alternative way to produce functional Cdk2-Speedy/Ringo A2 complexes, we also developed a coexpression system in which a C-terminally hexahistidine-tagged form of Speedy/Ringo A2 was coexpressed with untagged Cdk2 in *E. coli*. [unP]Cdk2-Speedy/Ringo A2-his_6_ (K2A2^coexp^) was purified on a metal affinity column and by gel filtration chromatography. Fractions were resolved by 10% SDS-PAGE and transferred to a PVDF membrane. Total proteins were detected by staining with Coomassie Brilliant Blue R-250 (Fig. 1C *top panel*) and Cdk2 was detected by immunoblotting with anti-Cdk2 antibodies (Fig. 1C *lower panel*). Speedy/Ringo A2-his_6_, which migrated as a 42 kDa protein on 10% SDS-PAGE, co-eluted with Cdk2 from the gel filtration column. The apparent MW for the peak fractions (fractions 14–15) was ~80 kDa, close to the combined molecular weights of monomeric Cdk2 (33 kDa) and Speedy/Ringo A2-his_6_ (42 kDa). The diffuse appearance of Speedy/Ringo A2 is probably due to phosphorylation of Speedy/Ringo A2 by Cdk2 (data not shown). The ratio of Cdk2 to Speedy/Ringo A2-his_6_ appears to be close to 1:1 in fractions 14 and 15, suggesting that one molecule of Speedy/Ringo A2 binds one molecule of Cdk2. K2A2^coexp ^displayed very similar protein kinase activity to *in vitro*-assembled Cdk2-Speedy/Ringo A2 complexes (see below), suggesting that both types of complexes may be fully active. Note that because of the coexpression of Cdk2 and Speedy/Ringo A2, and the poor phosphorylation of Cdk2 bound to Speedy/Ringo A2 by both metazoan CAK (Cdk7/cyclinH/Mat1) and budding yeast Cak1p (see below), we could only produce the unphosphorylated form of K2A2^coexp^. This [unP]Cdk2-Speedy/Ringo A2 heterodimer was used as a control throughout these studies.

We measured the ATPase activities of Cdk2-cyclin A and Cdk2-Speedy/Ringo A2 to assess the relative fractions of functional complexes. We reasoned that ATPase activities would be less subject to substrate specificity effects conferred by cyclin A or Speedy/Ringo A2 than protein kinase assays. The ATPase activity of monomeric [unP]Cdk2 is low; both activating phosphorylation and cyclin-binding increase the turnover of ATP by 20–25 fold [29,30]. Therefore, ATPase activity should be a reasonable indicator for the adoption of the catalytically active conformation of Cdk2 in Cdk2-Speedy/Ringo A2 complexes. We determined the ATPase activities of [pT160]Cdk2-cyclin A and K2A2^coexp^. A time course experiment (Fig. 1D) showed that the rate of ATP hydrolysis remained linear during the assay. The relative ATPase activities of [pT160]Cdk2-cyclin A and K2A2^coexp ^were calculated from the slopes in Fig. 1D and are shown in Fig. 1E. The ATPase activity of K2A2^coexp ^was slightly higher than that of [pT160]Cdk2-cyclin A, indicating that Cdk2 in K2A2^coexp ^is as active as that in [pT160]Cdk2-cyclin A. We should emphasize that *in vitro*-assembled [unP]Cdk2-Speedy/Ringo A2 and K2A2^coexp ^displayed very similar activities and substrate specificities (see below, and data not shown). Thus, it appears that *in vitro*-assembled Cdk2-Speedy/Ringo A2 complexes are fully active and it is reasonable to assume that they will display physiological biochemical characteristics.

### Substrate specificity of Cdk2-Speedy/Ringo A2

**Figure 2:**
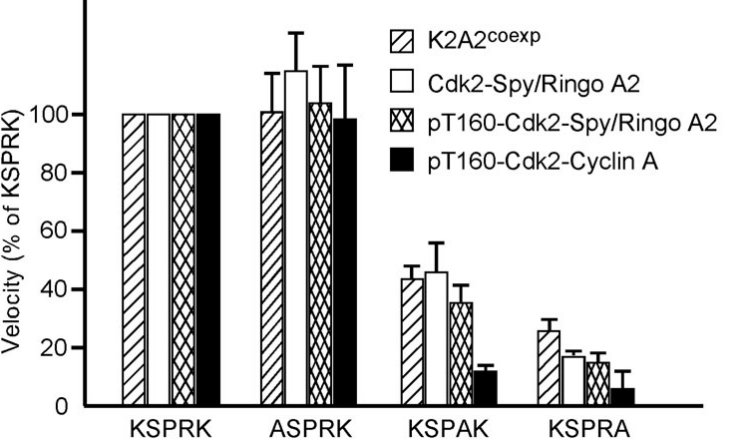
**Effects of alanine substitutions at each of the three charged positions in a KSPRK substrate on relative phosphorylation by Cdk2-cyclin A and Cdk2-Speedy/Ringo A2**. The phosphorylation efficiencies of the indicated GST peptides by K2A2^coexp^, [unP]Cdk2-Speedy/Ringo A2, [pT160]Cdk2-Speedy/Ringo A2, and [pT160]Cdk2-cyclin A were compared. Assays were performed at substrate concentrations of 50 μM. All values are relative to the phosphorylation of the wild type (KSPRK) substrate by the same enzyme. Values represent the means ± S.E. from three separate experiments.

We used a systematic panel of CDK substrates to investigate the substrate specificity of Cdk2-Speedy/Ringo A2 complexes. These substrates contained a pentapeptide of the form XSPXX (where X indicates any amino acid) fused to the C-terminus of GST [24]. Substrates were purified as GST-fusion proteins from *E. coli*. The optimal substrates for [pT161]Cdc2-cyclin B and [pT160]Cdk2-cyclin A have been identified as (K/R)(S/T)PX(K/R). Substitutions of the basic residue at the +3 position (with respect to the phosphorylation site) had the greatest effects on phosphorylation efficiencies, both by Cdc2 and by Cdk2 [24]. Thus, KSPRK is considered the "wild-type" substrate in these studies. We determined which position was most important for phosphorylation by Cdk2-Speedy/Ringo A2 using alanine substitution substrates in which the charged residues at -1, +2, and +3 were individually replaced with alanine. We compared the abilities of these substrates to be phosphorylated by Cdk2-Speedy/Ringo A2 (assembled *in vitro*), K2A2^coexp^, [pT160]Cdk2-Speedy/Ringo A2, and [pT160]Cdk2-cyclin A (Fig. 2). (Note that we are comparing relative substrate preferences and not absolute phosphorylation efficiencies, which vary greatly between Cdk2 bound to cyclin A and to Speedy/Ringo A2 (see below and Table 1).) For all of these enzymes, replacement of the lysine at the -1 position (ASPRK) had no effect on phosphorylation efficiency, substitution at the +2 position (KSPAK) had a modest effect, and substitution at the +3 position (KSPRA) produced the severest effects (Fig. 2). Cdk2-cyclin A was much more sensitive to the +2 and +3 substitutions than any of the forms of Cdk2-Speedy/Ringo A2. Similar effects were observed using K2A2^coexp ^and [unP]Cdk2-Speedy/Ringo A2, suggesting that the different ways in which these complexes were produced had little effect on their resulting substrate specificities. Phosphorylation of Cdk2 in Cdk2-Speedy/Ringo A2 complexes had virtually no effect on substrate specificity, which was surprising given the role of this phosphate in recognition of the +3 position of substrate by Cdk2-cyclin A [30,31]. Nevertheless, the +3 position was the most important position for both [unP]Cdk2-Speedy/Ringo A2 and [pT160]Cdk2-Speedy/Ringo A2, just as it is for [pT160]Cdk2-cyclin A.

**Table 1:** *Comparison of the relative phosphorylation efficiencies of the indicated substrates by [pT160]Cdk2-cyclin A, [unP]Cdk2-Speedy/Ringo A2, [pT160]Cdk2-Speedy/Ringo A2, and coexpressed [unP]Cdk2-Speedy/Ringo A2 *(K2A2^coexp^). Values represent the means from three independent experiments.

Enzyme complex	Histone H1 (5 μM)	KSPRK (50 μM)	KSPRR (50 μM)	KSPRY (50 μM)
	(pmol phosphate·min^-1^·μg^-1 ^Cdk2)			
[pT160]Cdk2-cyclin A	1983	1062	90	3.3
[unP]Cdk2- Speedy/Ringo A2	1.68	0.83	2.55	2.43
[pT160]Cdk2- Speedy/Ringo A2	2.05	1.02	3.13	0.68
K2A2^coexp^	1.55	1.40	5.5	3.15

**Figure 3:**
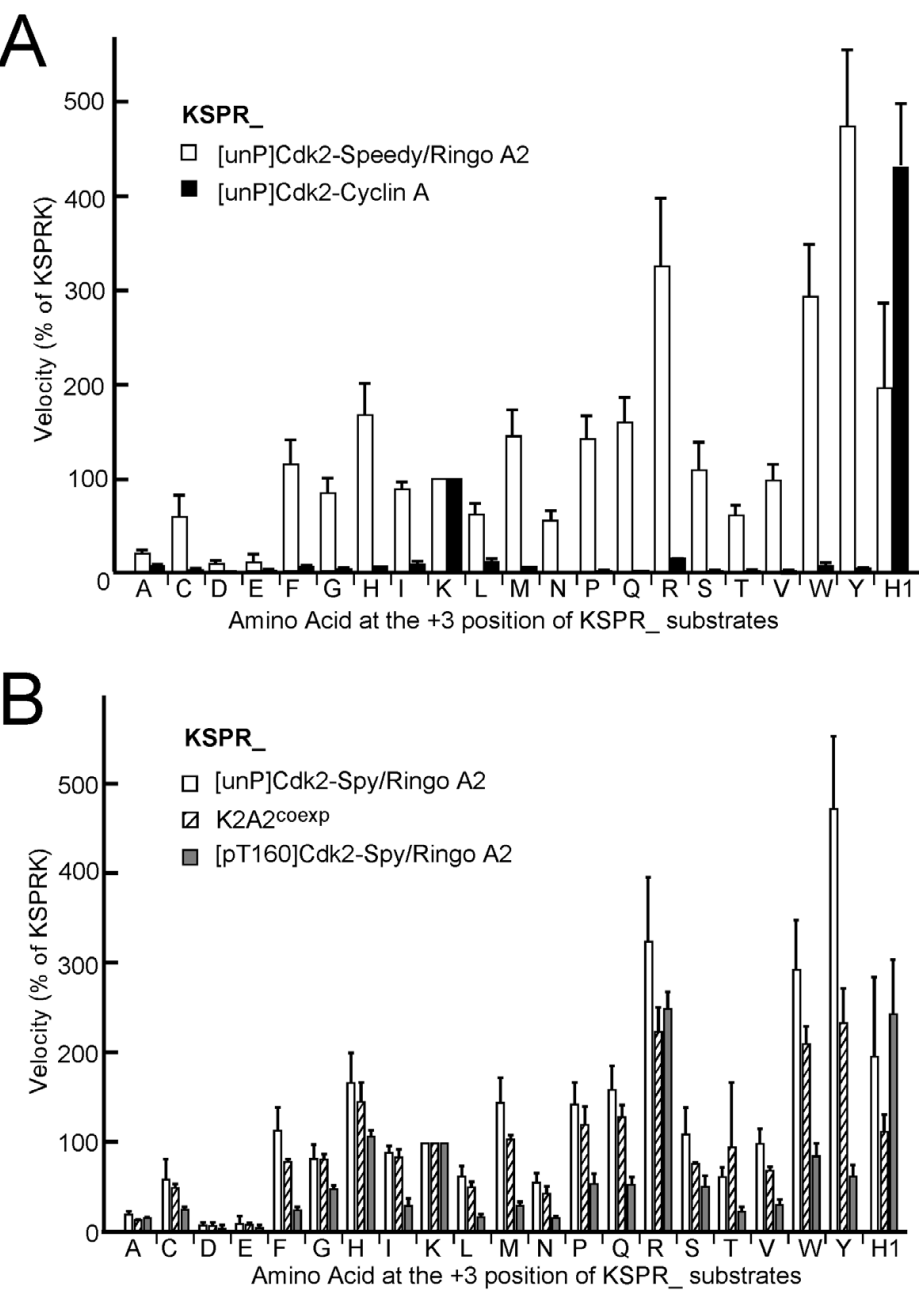
**Effects of amino acid substitutions at the +3 position of KSPRK on substrate utilization by [unP]Cdk2-Speedy/Ringo A2, [unP]Cdk2-cyclin A, K2A2^coexp^, and [pT160]Cdk2-cyclin A**. (A) Comparison of the substrate specificity of [unP]Cdk2-Speedy/Ringo A2 (open bars) and [unP]Cdk2-cyclin A (solid bars). Assays were performed at substrate concentrations of 50 μM. All phosphorylation efficiencies are relative to the phosphorylation of the KSPRK substrate by the same enzyme. Values represent the means ± S.E. from three separate experiments. (B) Comparison of the substrate specificities of [unP]Cdk2-Speedy/Ringo A2, K2A2^coexp^, and [pT160]Cdk2-Speedy/Ringo A2. Assays were performed at substrate concentrations of 50 μM. All phosphorylation efficiencies are relative to the phosphorylation of the KSPRK substrate by the same enzyme. Values represent the means ± S.E. from three separate experiments. Single letters indicate the amino acid at the +3 position of KSPRK. H1, histone H1.

We next evaluated the substrate specificity of Cdk2-Speedy/Ringo A2 at the +3 position in more detail using a panel of GST-KSPRX substrates [24]. We first compared the substrate specificity of [unP]Cdk2-Speedy/Ringo A2 with that of [unP]Cdk2-cyclin A, allowing us to compare the effects of the binding partner, separate from effects due to the Cdk2 phosphorylation state. The relative specificities of [unP]Cdk2-Speedy/Ringo A2 and [unP]Cdk2-cyclin A were determined at a substrate concentration of 50 μM, which is well below the *KM *value of Cdk2-Speedy/Ringo A2 and [pT160]Cdk2-cyclin A (see below and [24]) for these substrates, and thus within the linear range of the assay. We also compared the abilities of these kinases to phosphorylate histone H1 (5 μM). The phosphorylation of the "wild-type" substrate (GST-KSPRK) was defined as 100%. Although [unP]Cdk2-cyclin A was less active than fully activated [pT160]Cdk2-cyclin A, [unP]Cdk2-cyclin A still preferred lysine and arginine residues at the +3 position (Fig. 3A), consistent with a previous report indicating that [unP]Cdk2-cyclin A was only moderately defective in substrate binding [29]. In striking contrast to [unP]Cdk2-cyclin A, [unP]Cdk2-Speedy/Ringo A2 tolerated nearly all amino acid substitutions at the +3 position (Fig. 3A). The best substrates for [unP]Cdk2-Speedy/Ringo A2 contained tyrosine (Y) at 473 ± 82%, arginine (R) at 325 ± 74%, and tryptophan (W) at 293 ± 56%. More than half of the amino acid substitutions at the +3 position produced better substrates than lysine. In fact, 17 out of the 20 substrates were phosphorylated at least 50% as efficiently as KSPRK; only alanine, asparagine, and glutamine yielded poor substrates. In contrast, [pT160]Cdk2-cyclin A was unable to phosphorylate any substitution substrate more than 5% as efficiently as KSPRK, and most substitutions produced substrates whose phosphorylation was undetectable (< 0.01% of KSPRK).

We next compared the sensitivities of *in vitro*-assembled [unP]Cdk2-Speedy/Ringo A2 and K2A2^coexp ^to substitutions at the +3 position to test whether the route of formation of these complexes affected their substrate utilization. As shown in Fig. 3B, *in vitro*-assembled [unP]Cdk2-Speedy/Ringo A2 and K2A2^coexp ^exhibited almost identical phosphorylation efficiency profiles for the tested substrates, including histone H1. Except for the KSPRK and KSPRT substrates, K2A2^coexp ^phosphorylated all substrates slightly less efficiently than *in vitro*-assembled [unP]Cdk2-Speedy/Ringo A2, possibly due to its slightly higher enzymatic activity toward KSPRK (Table I), which was defined as 100%. Overall, the differences in substrate specificity and enzymatic activity between the *in vitro*-assembled complexes and K2A2^coexp ^were marginal. Both complexes could accept any amino acid side chain except aspartate and glutamate at the +3 position.

We then determined the effect of Thr-160 phosphorylation on the substrate specificity of [pT160]Cdk2-Speedy/Ringo A2. The optimal substrates at the +3 position were arginine (R) at 250 ± 19%, histidine (H) at 107 ± 6%, and lysine (K) at 100%. Phosphorylation of KSPRW and KSPRY were reduced from the very high levels exhibited by [unP]Cdk2-Speedy/Ringo A2 to levels more typical of other amino acid substitutions. Overall, Thr-160 phosphorylation modulated the substrate specificity of Cdk2-Speedy/Ringo A2 but did not transform it into a dramatically different pattern (Fig. 3B).

To determine the absolute (rather than relative) activities of Cdk2-cyclin A and Cdk2-Speedy/Ringo A2, we compared the abilities of [pT160]Cdk2-cyclin A and Cdk2-Speedy/Ringo A2 to phosphorylate several substrates, including histone H1, KSPRK, KSPRR, and KSPRY (Table I). For Cdk2-cyclin A, histone H1 and KSPRK are the best substrates, although KSPRR also fits the consensus substrate sequence, and KSPRY is an unfavorable substrate [24]. Cdk2-Speedy/Ringo A2 phosphorylated all four substrates with similar efficiencies (Table 1). [unP]Speedy/Ringo A2, [pT160]Cdk2-Speedy/Ringo A2, and K2A2^coexp ^phosphorylated the optimal Cdk2-cyclin A substrates histone H1 and KSPRK only about 0.1% as efficiently as [pT160]Cdk2-cyclin A (Table 1). In fact, the Cdk2-Speedy/Ringo A2 complexes were only about 6-fold more active toward histone H1 than monomeric [pT160]Cdk2 (Fig. 1B). The differences between Cdk2-cyclin A and Cdk2-Speedy/Ringo A2 decreased dramatically when activity was measured toward poorer Cdk2-cyclin A substrates. For instance, K2A2^coexp ^phosphorylated KSPRR 6% as efficiently as [pT160]Cdk2-cyclin A and it phosphorylated KSPRY 95% as efficiently as [pT160]Cdk2-cyclin A (Table I). Thus, the relative activities of Cdk2 bound to cyclin A or to Speedy/Ringo A2 depend critically on the substrate used.

**Figure 4:**
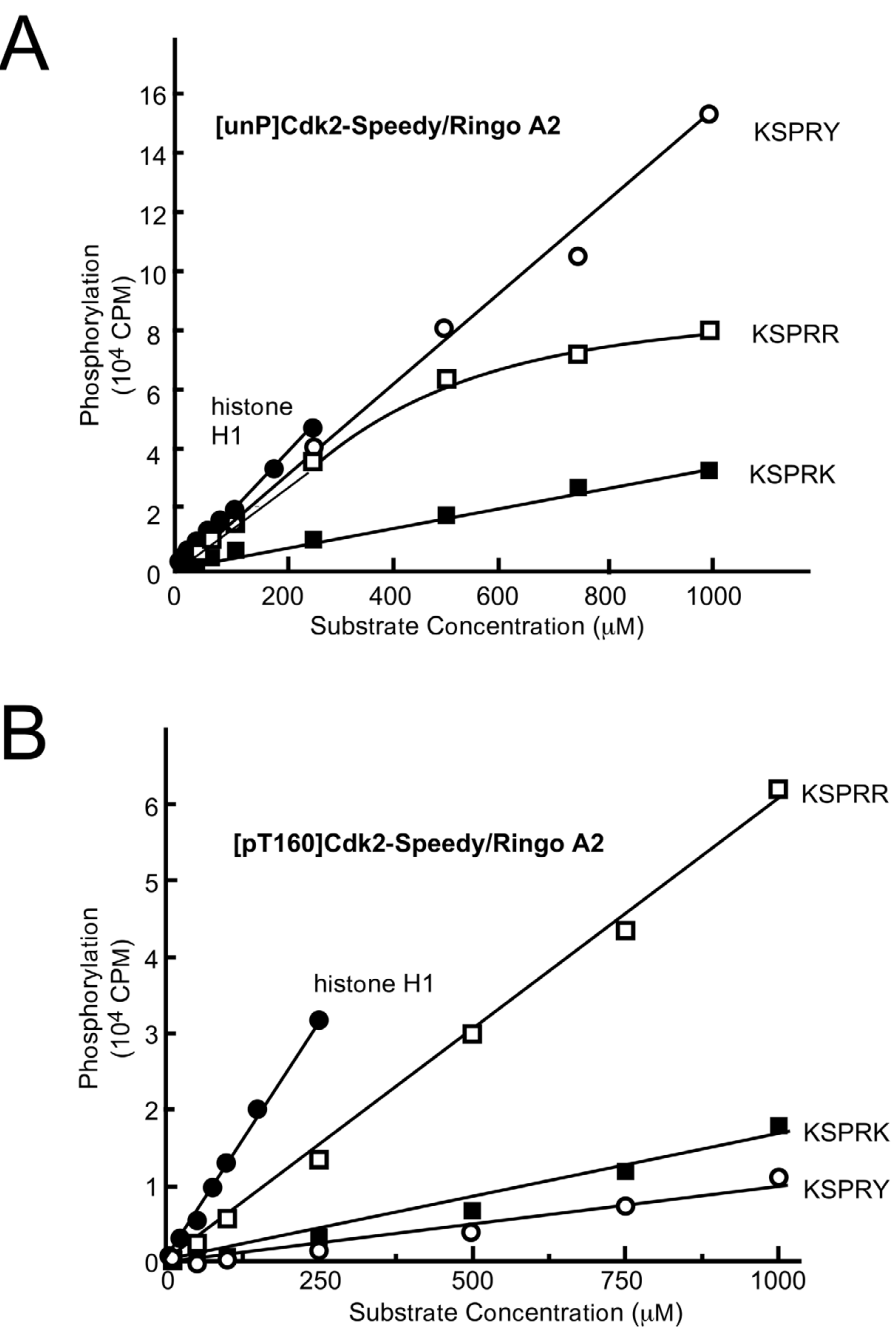
**Velocity *versus *concentration plots for phosphorylation of substrates by [unP]Cdk2-Speedy/Ringo A2 and [pT160]Cdk2-Speedy/Ringo A2**. Velocity *versus *concentration plots for [unP]Cdk2-Speedy/Ringo A2 (A) and [pT160]Cdk2-Speedy/Ringo A2 (B). The substrates are histone H1 (solid circles), KSPRK (solid squares), KSPRR (open squares), and KSPRY (open circles).

To understand why Cdk2-Speedy/Ringo A2 was much less active than [pT160]Cdk2-cyclin A toward certain substrates, we tried to assess the *K_M _*and catalytic activity (*V_max_*) of Cdk2-Speedy/Ringo A2. We chose several substrates including KSPRK, KSPRR, KSPRY, and histone H1 for this analysis. Phosphorylation of these substrates by [unP]Cdk2-Speedy/Ringo A2 and [pT160]Cdk2-Speedy/Ringo A2 was carried out over a wide range of substrate concentrations. The maximal concentrations were 250 μM for histone H1 and 1000 μM for the GST-peptide substrates. The phosphorylation of histone H1, KSPRY, and KSPRK by [unP]Cdk2-Speedy/Ringo A2 increased linearly up to the maximum concentration of each substrate (Fig. 4A). Interestingly, the phosphorylation of the KSPRR substrate plateaued at high substrate concentrations (open squares in Fig. 4A). We observed a similar phosphorylation efficiency-concentration relationship using K2A2^coexp ^(data not shown), suggesting that KSPRR is a good substrate for [unP]Cdk2-Speedy/Ringo A2. The phosphorylation of KSPRY (open circles) continued to increase linearly even at substrate concentrations at which the phosphorylation of KSPRR had plateaued, indicating that [unP]Cdk2-Speedy/Ringo A2 might have a stronger affinity for the KSPRR peptide but that it can perform the chemical steps of catalysis more efficiently on KSPRY. We also determined the utilization of KSPRK, KSPRR, KSPRY, and histone H1 by [pT160]Cdk2-Speedy/Ringo A2 over the same range of substrate concentrations. The phosphorylation of these four substrates increased linearly throughout the concentration range (Fig. 4B). Surprisingly, the phosphorylation of KSPRR by [pT160]Cdk2-Speedy/Ringo A2 increased linearly throughout the concentration range, suggesting that activating phosphorylation actually decreased the affinity of Cdk2-Speedy/Ringo A2 for KSPRR. These results differ significantly from those obtained using Cdk2-cyclin A, which has a *K*_*M *_for phosphorylation of histone H1 of 0.8 μM [29], and for phosphorylation of KSPRK of 150 μM [24]. Thus, it appears that weak substrate binding contributed, at least partially, to the low enzymatic activity of Cdk2-Speedy/Ringo A2 toward these substrates.

### Phosphorylation of Cdc25 proteins

The above results indicate that Cdk2-Speedy/Ringo A2 complexes phosphorylate CDK substrates with canonical motifs (S/T)PX(K/R) poorly compared to Cdk2-cyclin A, but that they can phosphorylate non-canonical CDK substrates relatively well. These findings suggested that Cdk2-Speedy/Ringo A2 might efficiently phosphorylate some physiological Cdk2 substrates containing non-canonical motifs. We reasoned that Cdc25 proteins might be such substrates. Autoamplification of Cdc2 activity at the G2/M transition was proposed more than a decade ago [8]. In this model, Cdc25 protein phosphatases activate Cdc2-cyclin B complexes by removing inhibitory phosphates from Cdc2. In turn, active Cdc2-cyclin B phosphorylates and activates Cdc25, which activates additional Cdc2-cyclin B complexes. Thus, a low level of CDK activity can be amplified through this positive feedback loop, leading to the concerted activation of Cdc2 and entry into mitosis. To achieve an abrupt all-or-none activation of Cdc2 at the right time, it is important that Cdc25 proteins not be activated at too low a level of Cdc2 activity. From this point of view, one would predict that Cdc25 would be a poor CDK substrate. A similar analysis applies to the activation of Cdk2 complexes by Cdc25 proteins.

**Figure 5:**
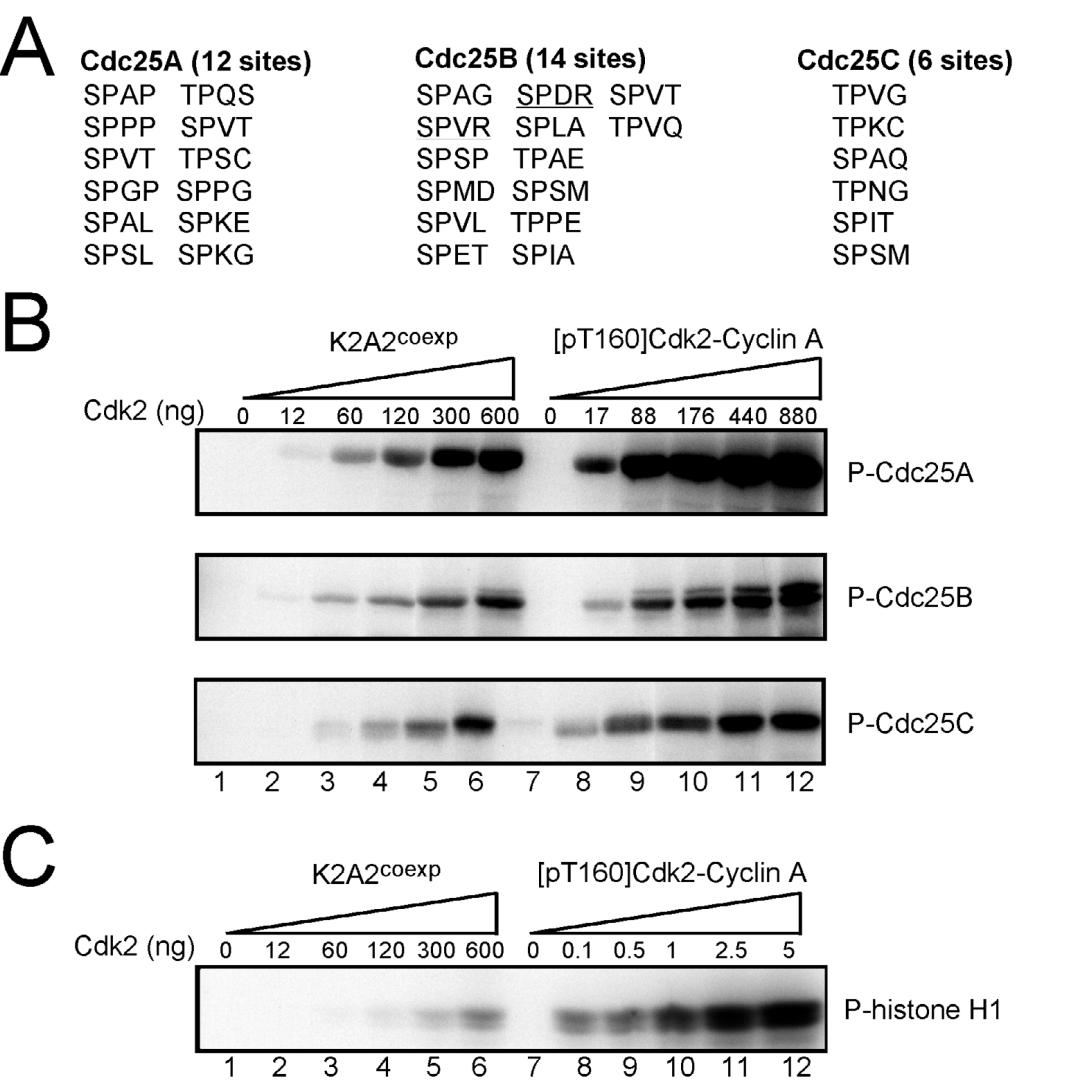
**Phosphorylation of Cdc25A, B, and C by Cdk2-Speedy/Ringo A2**. (A) Potential CDK phosphorylation sites in human Cdc25A, B, and C. There are 12 (S/T)PXX sequences in Cdc25A, 14 in Cdc25B, and 6 in Cdc25C. The two sequences that come closest to fitting the consensus CDK phosphorylation sequence (S/T)PX(K/R) are underlined. (B) Phosphorylation of Cdc25A, B, and C by K2A2^coexp ^and [pT160]Cdk2-cyclin A. Phosphorylation of GST-Cdc25A, B, and C (5 μg) by the indicated amounts of K2A2^coexp ^(*lanes 1–6*) or Cdk2-cyclin A (*lanes 7–12*). Phosphorylated proteins were separated by SDS-PAGE and detected by autoradiography. (C) Phosphorylation of histone H1 by K2A2^coexp ^and [pT160]Cdk2-cyclin A. Histone H1 (5 μM) was phosphorylated by the indicated amounts of K2A2^coexp ^(*lanes 1–6*) or Cdk2-cyclin A (*lanes 7–12*). Phosphorylated proteins were separated by SDS-PAGE and detected by autoradiography.

All three mammalian Cdc25 isoforms (A, B, and C) can be phosphorylated on multiple sites by CDK-cyclin complexes (reviewed in [35-37]). Among 32 potential CDK phosphorylation sites ((S/T)PXX) in human Cdc25A, B, and C (Fig. 5A), only 2 sites fit the consensus CDK phosphorylation motif, and even these are weak fits for phosphorylation by Cdk2 (SPXR) since they lack a lysine at the +3 position [24]. We conclude that most CDK phosphorylation of Cdc25 proteins occurs on non-canonical motifs. We, therefore, examined whether Cdc25 proteins could be phosphorylated by Cdk2-Speedy/Ringo A2. As shown in Fig. 5B, both Cdk2-cyclin A and K2A2^coexp ^phosphorylated GST-Cdc25A, B, and C. To compare the activities of Cdk2-cyclin A and Cdk2-Speedy/Ringo A2 toward Cdc25, we varied the amounts of [pT160]Cdk2-cyclin A and K2A2^coexp ^used to phosphorylate constant amounts of GST-Cdc25A, B, and C. We estimated that the enzymatic activity of [pT160]Cdk2-cyclin A toward the Cdc25 proteins was 7–14 fold higher than that of K2A2^coexp^. In contrast, [pT160]Cdk2-cyclin A phosphorylated histone H1 >1000-fold more efficiently than Cdk2-Speedy/Ringo A2 (Fig. 5C).

**Figure 6:**
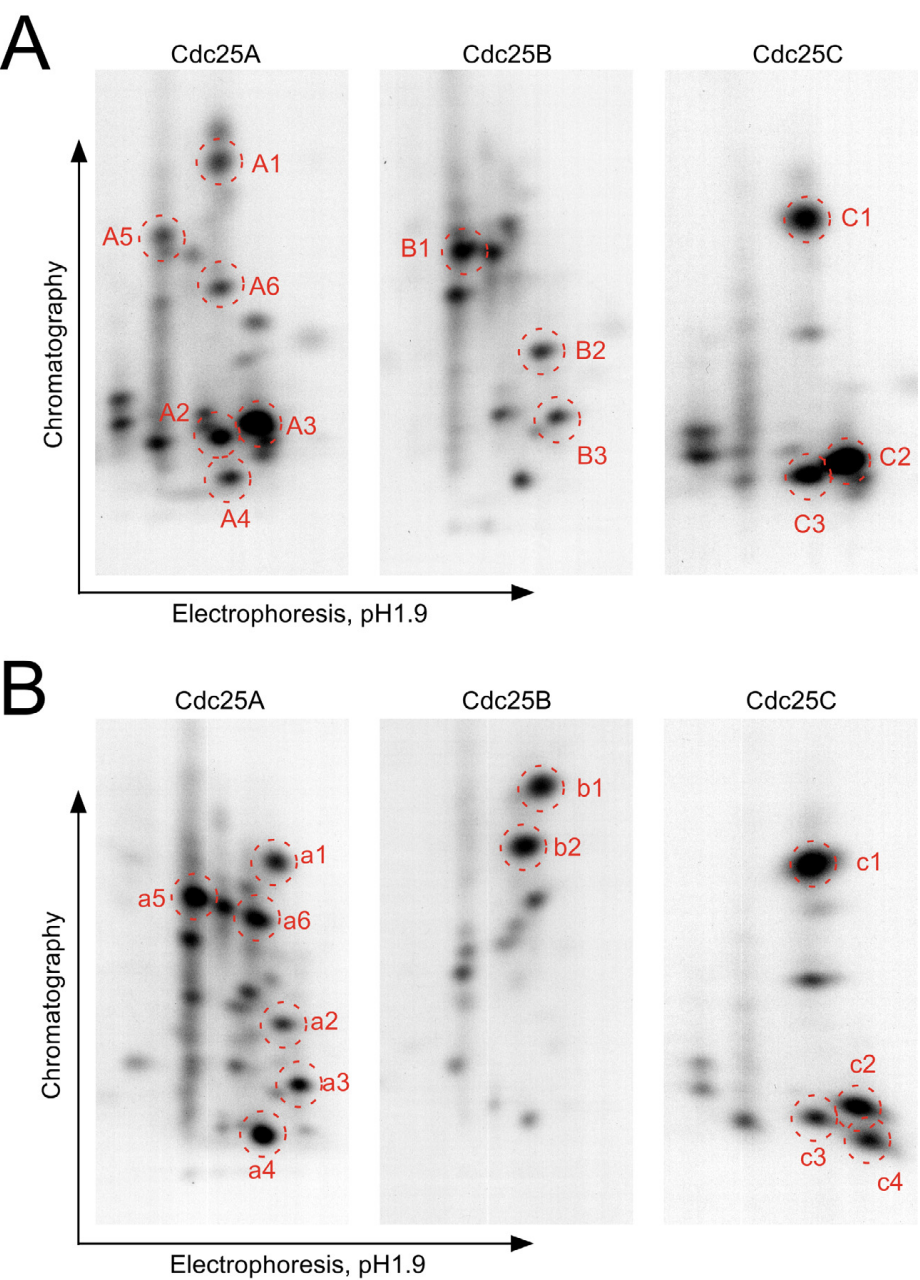
**Trypic phosphopeptide mapping of Cdc25 proteins**. Tryptic phosphopeptide mapping of Cdc25 phosphorylated by [pT160]Cdk2-cyclin A (A) and by K2A2^coexp ^(B). GST-Cdc25A, B, and C (5 μg) were phosphorylated by K2A2^coexp ^and [pT160]Cdk2-cyclin A, separated by 10% SDS-PAGE, extracted, and digested with trypsin. Phosphopeptides were separated on thin layer chromatography plates by electrophoresis followed by chromatography and were detected by autoradiography.

To determine whether Cdk2-Speedy/Ringo A2 and Cdk2-cyclin A phosphorylate Cdc25 proteins on the same or different sites, we compared tryptic phosphopeptide maps of Cdc25 proteins phosphorylated by [pT160]Cdk2-cyclin A (Fig. 6A) with those phosphorylated by K2A2^coexp ^(Fig. 6B). Each Cdc25 protein was phosphorylated to about the same extent by each Cdk2 complex. Since there are multiple potential CDK phosphorylation sites in Cdc25 proteins (Fig. 5A), it was not surprising that multiple sites were actually phosphorylated. As shown in Fig. 6, phosphorylation of each Cdc25 protein by Cdk2-Speedy/Ringo A2 and by Cdk2-cyclin A produced distinct phosphopeptide patterns. These phosphopeptides could be grouped into two categories. First, many phosphopeptides were unique for either Cdk2-cyclin A or Cdk2-Speedy/Ringo A2, indicating a very strong influence of Cdk2 partner on phosphorylation specificity. For example, spots A1, A2, B1, B2, and B3 were unique for Cdk2-cyclin A, whereas spots a1, a2, b1, b2, and c4 were exclusively associated with Cdk2-Speedy/Ringo A2. Phosphopeptides in the second group were phosphorylated by both kinases, but to different extents. For example, in the phosphopeptide maps of Cdc25A, spots A3, A4, A5, and A6 generated by Cdk2-cyclin A (Fig. 6A) appear to be identical to spots a3, a4, a5, and a6 generated by Cdk2-Speedy/Ringo A2 (Fig. 6B), respectively. While Cdk2-cyclin A phosphorylated site A3 more strongly than sites A4, A5, and A6, Cdk2-Speedy/Ringo A2 had the opposite site preference. In the phosphopeptide maps of Cdc25C, Cdk2-cyclin A phosphorylated peptide C2 more efficiently than peptide C1 whereas Cdk2-Speedy/Ringo A2 phosphorylated C1 more efficiently than C2. Therefore, Cdk2-Speedy/Ringo A2 and Cdk2-cyclin A have distinct but partially overlapping substrate preferences on natural substrates.

### Cdk2 activation loop conformation in Cdk2-Speedy/Ringo A2 complexes

We used accessibility to kinases and phosphatases to probe the conformation of the Cdk2 activation loop in Cdk2-Speedy/Ringo A2 complexes. The activation loop plays an important role in substrate recognition by Cdk2-cyclin A as the Thr-160 phosphate makes direct contact with the +3 position of substrates. The strikingly different substrate preferences of Cdk2-cyclin A and Cdk2-Speedy/Ringo A2 raised the possibility that the activation loop in Cdk2-Speedy/Ringo A2 might adopt a different conformation from that exhibited by Cdk2-cyclin A. In addition, like Cdk2-Speedy/Ringo A2, monomeric [pT160]Cdk2 displayed low enzymatic activity toward histone H1 ([29,33] and Fig. 1B) and defective substrate binding [29]. Structural studies indicate that the activation loop in monomeric [pT160]Cdk2 is highly disorganized and that only a very small population of [pT160]Cdk2 is in the active conformation at any given time [33]. This comparison raised the possibility that the activation loop in Cdk2-Speedy/Ringo A2 might also be disordered.

**Figure 7:**
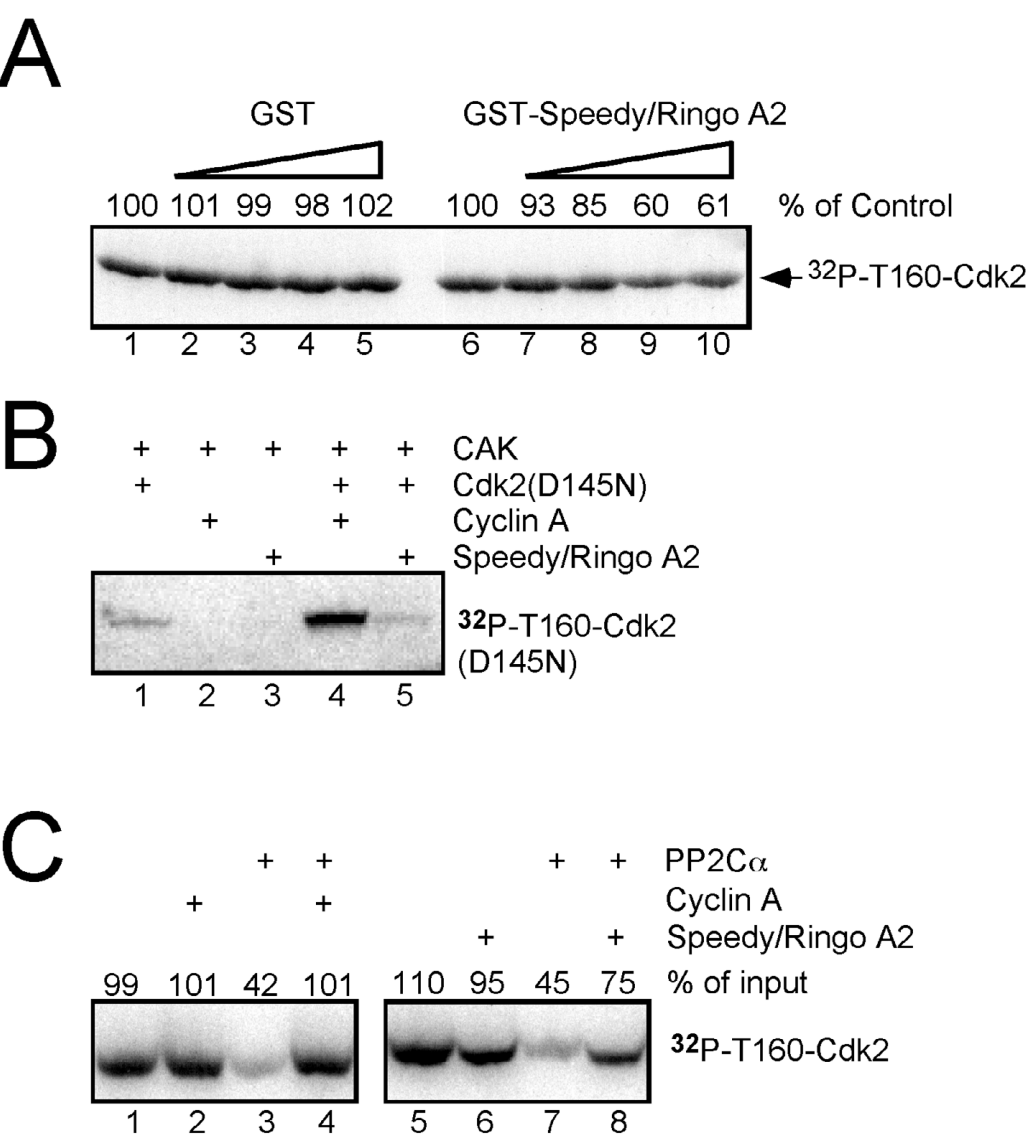
**Effects of Speedy/Ringo A2 binding on the phosphorylation and dephosphorylation of Cdk2 on Thr-160**. (A) Speedy/Ringo A2 hinders the phosphorylation of Cdk2 on Thr-160 by budding yeast Cak1p. GST-Cdk2^D145N ^(1 μg) was incubated with increasing amounts (0, 1, 2.5, 5, and 10 μg) of GST-Speedy/Ringo A2 or GST prior to phosphorylation by Cak1p (100 ng). The reactions were terminated and phosphorylated proteins were resolved by SDS-PAGE and detected by autoradiography. (B) Speedy/Ringo A2 binding did not stimulate Thr-160 phosphorylation by mammalian CAK. GST-Cdk2^D145N ^(1 μg; *lanes 1, 4, 5*) was preincubated with cyclin A (1 μg; *lanes 2 *and *4*) or GST-Speedy/Ringo A2 (4 μg; *lanes 3 *and *5*) before phosphorylation by CAK. Samples were processed as described in *Methods*. (C) Speedy/Ringo A2 hinders the dephosphorylation of Cdk2 on Thr-160 by PP2Cα. [^32^P-T160]GST-Cdk2^D145N ^was preincubated with cyclin A (*lanes 2 *and *4*), GST-Speedy/Ringo A2 (*lanes 6 *and *8*), or buffer alone (*lanes 1, 3, 5, 7*) prior to addition of recombinant human PP2Cα (*lanes 3, 4, 7, 8*) or buffer (*lanes 1, 2, 5, 6*). [^32^P-T160]GST-Cdk2^D145N ^was detected by autoradiography.

We first probed the T-loop conformation in Cdk2-Speedy/Ringo A2 using budding yeast Cak1p. Cak1p phosphorylates monomeric Cdk2 efficiently, but this phosphorylation is inhibited over 95% in the presence of cyclin A [38], presumably because the fixed conformation of the activation loop in Cdk2-cyclin A forms a poor substrate for Cak1p. A kinase-inactive form of Cdk2 (GST-Cdk2^D145N^) was used in this assay to eliminate the high background phosphorylation of Speedy/Ringo A2 by wild-type Cdk2. GST-Cdk2^D145N ^bound *in vitro *translated [^35^S]-Speedy/Ringo A2 as well as wild-type Cdk2 (data not shown). GST-Cdk2 was preincubated with increasing amounts of GST-Speedy/Ringo A2 or GST alone before phosphorylation by Cak1p. As shown in Fig. 7A, the phosphorylation of Cdk2 by Cak1p was reduced in the presence of high concentrations of Speedy/Ringo A2, indicating that the binding of Speedy/Ringo A2 to Cdk2 changed the conformation of the activation loop.

We next investigated the T-loop conformation of Cdk2-Speedy/Ringo A2 using mammalian CAK (Cdk7/cyclin H/Mat1). Monomeric CDKs are poor substrates for mammalian CAK whereas the binding of cyclin stimulates activating phosphorylation by CAK more than seven fold [38]. We again used a kinase-inactive form of GST-Cdk2 (GST-Cdk2^D145N^) to reduce background phosphorylation of GST-Speedy/Ringo A2 by wild-type Cdk2. GST-Cdk2^D145N ^alone was slightly phosphorylated by CAK (Fig. 7B, *lane 1*). The phosphorylation of GST-Cdk2^D145N ^was greatly enhanced in the presence of cyclin A (Fig. 7B, *lane 4*). In contrast, GST-Speedy/Ringo A2 had no effect on the phosphorylation GST-Cdk2^D145N ^by CAK (Fig. 7B, *lane 5*). The defective phosphorylation of Cdk2-Speedy/Ringo A2 by mammalian CAK is unlikely to be due to a failure of Speedy/Ringo A2 and GST-Cdk2^D145N ^to form a complex. Taking these results together, we conclude that binding of Speedy/Ringo A2 alters the activation loop conformation of Cdk2 (Fig. 7A), but in a manner distinct from how cyclin A does so (Fig. 7B). Furthermore, it appears that Cdk2-Speedy/Ringo A2 is a poor substrate for mammalian CAK.

Finally, we examined the T-loop conformation in the Cdk2-Speedy/Ringo A2 complex using serine/threonine protein phosphatase type 2C (PP2C) [39]. Mammalian PP2Cα and β can remove the activating phosphate from monomeric CDKs such as Cdk2 and Cdk6 [40]. The binding of cyclin A to Cdk2 prevents dephosphorylation by PP2C [40], presumably because the activation loop becomes locked into a conformation inaccessible to PP2C [41]. We tested whether Speedy/Ringo A2, like cyclin A, can prevent dephosphorylation by PP2Cα. GST-Cdk2^D145N ^was labeled with [γ^-32^P]-ATP using Cak1p and purified by gel filtration. ^32^P-labeled Cdk2 was preincubated with Speedy/Ringo A2, cyclin A, or buffer before addition of PP2Cα. As described previously [40], cyclin A fully blocked the dephosphorylation of Cdk2 (Fig. 7C, compare *lanes 3 *and *4*). The binding of Speedy/Ringo A2 partially blocked the dephosphorylation of Cdk2 by PP2Cα (Fig. 7C, *lane 8*). Based on this intermediate result we conclude, as with the Cak1p and CAK results above, that binding to Speedy/Ringo A2 alters the conformation of the activation loop, but that this conformation differs from that seen after binding of cyclin A.

## Discussion

The recently discovered Speedy/Ringo proteins represent a novel class of non-cyclin CDK activators that play important roles in cell cycle progression. *Xenopus *Speedy/Ringo is necessary for G2/M progression during oocyte maturation and a human Speedy/Ringo protein (Spy1) regulates S-phase entry in cultured cells [25-28]. Although apparently not present in yeast, plants, and insects, Speedy/Ringo homologues can be found in the most primitive branching clade of chordates (*Ciona intestinalis*) [34], from which all vertebrates evolved. It is conceivable that Speedy/Ringo proteins regulate cell cycle progression in all vertebrates. Although there is no obvious primary sequence similarity between cyclins and Speedy/Ringo proteins, Speedy/Ringo proteins can bind to and activate CDKs directly [27,34]. In this study, we carried out a biochemical characterization of Cdk2-Speedy/Ringo A2 complexes. We verified that human Speedy/Ringo A2 can form a 1:1 complex with Cdk2 and that it can activate Cdk2 *in vitro*, even in the absence of phosphorylation of Cdk2 on Thr-160.

However, Speedy/Ringo A2 is not a simple replacement for certain cyclins; there are many important biochemical differences between cyclins and Speedy/Ringo proteins. First, Cdk2-Speedy/Ringo A2 displays a broad substrate specificity, which is very different from the narrow consensus CDK phosphorylation motif. Previous studies showed that Cdk2-cyclin A phosphorylated (K/R)(S/T)PX(K/R) sequences, with a strong preference for a lysine at the terminal position. We found, using a systematic peptide substrate panel, that the +3 position is also the most important residue for Cdk2-Speedy/Ringo A2 recognition. At the +3 position, the best substrates for Cdk2-cyclin A are KSPRK and KSPRR (~5% of KSPRK), which contain basic residues at the +3 position [24]. In contrast, the best substrates for Cdk2-Speedy/Ringo A2 contained tyrosine (Y), arginine (R), and tryptophan (W) in the +3 position, all of which carry bulky side chains. Furthermore, Cdk2-Speedy/Ringo A2 complexes could tolerate almost any amino acid residue at the +3 position and phosphorylated 17 of the 20 +3 substrates at least 50% as well as KSPRK. More than half of the +3 substitutions yielded substrates that were phosphorylated more efficiently than KSPRK. Only alanine, aspartate, and glutamate formed poor substrates, whose relative phosphorylation by Cdk2-Speedy/Ringo A2 was still higher than the relative phosphorylation of all but a few of the +3 substrates by Cdk2-cyclin A.

The second difference between cyclins and Speedy/Ringo A2 is that Cdk2-Speedy/Ringo A2 possesses low enzymatic activity toward conventional CDK substrates, indicating that Speedy/Ringo A2 is unlikely to be able to replace cyclins and promote the full range of Cdk2 substrate phosphorylation. For example, the activity of Cdk2-Speedy/Ringo A2 toward histone H1 was only ~0.1% the activity of [pT160]Cdk2-cyclin A in the standard histone H1 kinase assay. Therefore, it is unlikely that CDK-Speedy/Ringo can promote cell cycle progression by itself. The broad tolerance of Cdk2-Speedy/Ringo A2 for substitutions at the +3 position of substrates and its low activity toward conventional Cdk2 substrates may go hand in hand. The insensitivity of Cdk2-Speedy/Ringo A2 to phosphorylation of Thr-160 on Cdk2 may also contribute to its low activity toward conventional substrates as this phosphate plays a direct role in substrate recognition via interaction with +3 basic residues. Indeed, the activation loop conformation adopted by Cdk2 upon binding Speedy/Ringo A2 appears to differ significantly from that adopted upon cyclin A binding. Cdk2-Speedy/Ringo A2 also appeared to bind substrates, except for KSPRR, poorly, which may contribute to its low enzymatic activity.

Although it displays a low enzymatic activity toward conventional CDK substrates such as histone H1, Cdk2-Speedy/Ringo A2 actually phosphorylated non-canonical CDK substrates nearly as well as Cdk2-cyclin A. We extended this observation made using model substrates to physiological CDK substrates, the Cdc25 dual-specificity phosphatases. We found that Cdk2-Speedy/Ringo A2 could phosphorylate the three human Cdc25 proteins quite efficiently, about 1000 times more efficiently than would be expected based on the histone H1 kinase activity of Cdk2-Speedy/Ringo A2 and only about an order of magnitude less well than Cdk2-cyclin A. Furthermore, phosphopeptide mapping of Cdc25 proteins confirmed that the substrate specificity of Cdk2-Speedy/Ringo A2 overlaps with but is distinct from that of Cdk2-cyclin A. It should also be pointed out that, in addition to inherent substrate specificity, some CDKs are targeted to some of their substrates. For example, the S-phase cyclins, such as cyclin A and Clb5, possess a 'hydrophobic patch' that can interact with the 'RXL' or 'Cy' motif present in some substrates to carry out specific functions during DNA replication [18,31,42-46]. In contrast, phosphorylations of histone H1 and GST-peptide substrates are independent of any docking site. Thus, for Cdk2-Speedy/Ringo A2, the existence of a docking site on Speedy/Ringo A2 would greatly increase the phosphorylation of select substrates by Cdk2-Speedy/Ringo A2.

A third difference between cyclins and Speedy/Ringo A2 is that Speedy/Ringo A2 can activate Cdk2 independent of the activating phosphorylation on Thr-160 of Cdk2. Previous studies have shown that *Xenopus *Speedy/Ringo can activate Cdc2 and Cdk2 *in vitro *in the absence of activating phosphorylation and that it can render Cdk2 less sensitive to inhibition via inhibitory phosphorylation and binding of CKIs such as p21 [27]. We examined whether activating phosphorylation, though not necessary for phosphorylation of histone H1, might nonetheless affect the substrate specificity of Cdk2-Speedy/Ringo A2. Neither the overall catalytic activity of Cdk2-Speedy/Ringo A2 nor its substrate recognition required the activating phosphorylation of Cdk2. In fact, Thr-160 phosphorylation reduced the activity of Cdk2-Speedy/Ringo A2 toward most of the tested substrates and did not promote activity toward any particular substrate. We also found that Cdk2-Speedy/Ringo A2 is a poor substrate for metazoan CAK (Cdk7/Cyclin H/Mat1). These findings indicate that the activation of Cdk2 by Speedy/Ringo A2 does not require the activating phosphorylation by CAK and that it may proceed in the absence of this phosphorylation.

## Conclusion

We have identified crucial biochemical differences between Cdk2-cyclin A and Cdk2-Speedy/Ringo A2 complexes. These findings raise the possibility that Speedy/Ringo A2 could play a significant role in the phosphorylation of CDK substrates containing non-canonical phosphorylation sites and suggest that Cdc25 proteins might be physiological targets for Cdk2-Speedy/Ringo A2 complexes. This is an intriguing possibility given the positive feedback that exists, for instance, between activation of Cdc2 by Cdc25 and of Cdc25 by Cdc2. CDK-Speedy/Ringo complexes – which do not require activating phosphorylation by CAK and are less sensitive to inhibitory phosphorylation and CDK inhibitors such as p21 – would be in a strong position to jump-start a positive feedback loop or to reverse the inhibition of Cdc25 caused by stresses such as that caused by DNA damage.

## Methods

### Reagents

[γ^-32^P]-ATP (3000 Ci/mmol) was from Dupont-NEN (Boston, MA). *E. coli *BL21-Codon plus^RIL ^and BL21(DE3)-Codon plus^RIL ^cells were from Stratagene (La Jolla, CA). Calf thymus histone H1 (Cat. #1004875) was from Roche Diagnostics Inc (Indianapolis, IN). All other chemicals were from Sigma (St. Louis, MO) unless indicated otherwise. 1 × protease inhibitor mix (PI) contained 1 mM PMSF and 10 μg/ml each of leupeptin, chymostatin, and pepstatin. EB buffer is 80 mM β-glycerophosphate, pH 7.3, 20 mM EGTA, 15 mM MgCl_2_, 10 mM DTT, 1 mg/ml ovalbumin, and 1 × PI. Buffer A is 20 mM Tris-HCl, pH 7.4, 150 mM NaCl, 10 mM MgCl_2_, 1 mM DTT, 1 mg/ml ovalbumin, 0.1% Tween 20, 1 × protease inhibitors. 1 × TBS buffer is 50 mM Tris-HCl, pH 7.4, 150 mM NaCl, 0.5 mM EDTA, 0.5 mM DTT. Rabbit anti-Cdk2 antibodies were from Santa Cruz Biotech (Santa Cruz, CA). HRP-conjugated secondary antibodies and SuperSignal™ ECL reagents were from Pierce (Rockford, IL).

### Protein expression and purification

GST-Speedy/Ringo A2 [39], GST-Cdk2 [31], human PP2Cα [40], GST-Cdk2 mutants [38,47], GST-Cak1p [14], Cdk7/cyclin H [38], and GST fusion substrates [24] have been described previously. The GST-Cdk2/GST-Cak1p bicistron expression plasmid was constructed by Neil Hanlon (University of Oxford, UK) and provided by Louise Johnson (University of Oxford, UK). GST-[pT160]Cdk2 was expressed as described [31]. A plasmid expressing a C-terminally his_6_-tagged version of the portion of bovine cyclin A3 corresponding to residues 171–432 of human cyclin A was provided by John Lew (University of California, Santa Barbara, CA) and purified as described [48]. For simplicity, we refer to this protein as cyclin A. Expression vectors for GST-Cdc25A, B, and C were provided by Anindya Dutta (University of Virginia) [49].

To coexpress Cdk2-Speedy/Ringo A2-His_6_ in bacteria (K2A2^coexp^), mouse Speedy/Ringo A2 was first amplified by polymerase chain reaction using an N-terminal primer to incorporate a *Hin*dIII site and a ribosome binding site before the start codon, and a C-terminal primer to remove the stop codon and add a *Xho*I site. The DNA sequences of the PCR primers are as follows (cloning sites are underlined): 5'-CCCCAAGCTTAAGGAGGGATAGCCATGGGACGGCATAATCAGATGTATTG-3' and 5'-CCCCCTCGAGTTCTTCACTCTCTGCAAACC-3'. The resulting DNA was digested with the indicated restriction enzymes and cloned into pET21d (Novagen) at the *Hin*dIII and *Xho*I sites, placing it in-frame with a C-terminal polyhistidine (his_6_) tag in the vector. Finally, HA-tagged Cdk2 was excised from an expression vector [12] using *Nco*I and *Bam*HI and cloned into pET21d at the corresponding sites. The resulting K2A2^coexp ^coexpression vector was transformed into BL21(DE3)-Codon plus^RIL ^cells. K2A2^coexp ^was induced using IPTG, partially purified on a metal affinity column as described [39], and loaded onto a Superdex-200 column pre-equilibrated with 1 × TBS buffer at a flow rate of 0.5 ml/min. Fractions containing Cdk2 were pooled and concentrated to about 2 mg/ml. The concentration of Cdk2 was determined by immunoblotting using GST-Cdk2 as a standard. Cdk2-Speedy/Ringo A2 complexes produced by coexpression in bacteria are designated K2A2^coexp ^and always contained Cdk2 unphosphorylated on Thr-160.

Cdk2-Speedy/Ringo A2 complexes were also formed *in vitro *by mixing purified GST-Cdk2 with a three-fold molar excess of GST-Speedy/Ringo A2 in 1 × EB at room temperature for 20 min. For some experiments, the Cdk2 was first phosphorylated on Thr-160 by coexpression with Cak1p in bacteria (see above). Cdk2-cyclin A complexes were formed *in vitro *by mixing purified GST-[unP]Cdk2 or GST-[pT160]Cdk2 with an equal molar amount of cyclin A in 1 × EB at room temperature for 20 min.

### ATPase assays

To measure the rate of ATP hydrolysis by [pT160]Cdk2-cyclin A and K2A2^coexp^, 16.7 μM of [pT160]Cdk2-cyclin A or K2A2^coexp ^in 10 μl of ATPase buffer (50 mM Tris-HCl, pH 7.4, 15 mM MgCl_2_, 150 mM NaCl, 1 mg/ml ovalbumin, 10 mM DTT, 0.5% Tween-20, and 1 × protease inhibitors) was mixed with an equal volume of ATP mix containing 1 mM ATP and 0.5 μCi/μl [γ^-32^P]ATP in ATPase buffer. At each time point, 1 μl of the assay was mixed with 4 μl of Stop buffer (ATPase buffer containing 20 mM EDTA instead of 15 mM MgCl_2_). 1 μl of the terminated reaction was spotted onto a polyethyleneimine cellulose plate (Selecto Scientific, Norcross, GA), and chromatographed for 2 h in 50 mM HCl. Plates were dried and rechromatographed prior to PhosphorImager analysis.

### In vitro kinase assay and data analysis

Cdk2-Speedy/Ringo A2 mixtures were prepared as described above. The Cdk2 concentration in the enzyme mixture was adjusted to 0.1 μg/μl for GST-Cdk2 in GST-Cdk2-Speedy/Ringo A2 complexes and to 0.055 μg/μl for Cdk2 in K2A2^coexp^. To determine the substrate specificity and enzymatic activity of Cdk2-Speedy/Ringo A2 (Figs. 2, 3), kinase assays were carried out by incubating 5 μl of enzymes (8.6 pmol of Cdk2) with 5 μl of substrates (13 μg GST substrates or 1.3 μg histone H1) in the presence of 0.25 μCi/μl [γ^-32^P]-ATP, and 0.4 mM ATP in 1 × EB at 25°C. Reactions proceeded for 10 min and were terminated by addition of 5 μl of 3 × SDS-PAGE sample buffer. Samples were resolved by 10% SDS-PAGE and analyzed by autoradiography and phosphorimaging. Substrate bands were also excised and quantified by liquid scintillation counting. To determine the phosphorylation efficiency-concentration plot (Fig. 4), 5 μl of enzymes (8.6 pmol) were incubated with 5 μl of substrates in the presence of 0.25 μCi/μl [γ^-32^P]-ATP, and 1 mM ATP in 1 × EB for 10 min at 25°C. The reaction was terminated by the addition of 5 μl of 3 × SDS-PAGE sample buffer. Samples containing more than 10 μg of GST substrates or 1 μg of histone H1 were diluted in 1 × sample buffer before SDS-PAGE. Samples were resolved by 10% SDS-PAGE and analyzed by autoradiography and phosphorimaging. Individual substrate bands were excised and quantified by liquid scintillation counting. Data were analyzed using the MS-excel program.

### Phosphorylation of GST-Cdc25A, B, C, and histone H1

For the comparative analysis of Cdc25 and histone H1 phosphorylation by Cdk2-Speedy/Ringo A2 and [pT160]Cdk2-cyclin A (Fig. 5), 5 μl of substrate (5 μg of GST-Cdc25 or 5 μM of histone H1 in EB) was mixed with 5 μl of enzyme mix containing the indicated amounts of coexpressed Cdk2-Speedy/Ringo A2 or of [pT160]Cdk2-cyclin A in the presence of 0.25 μCi/μl [γ^-32^P]-ATP, 0.4 mM ATP in 1 × EB. The reactions proceeded for 10 min at room temperature and were terminated by addition of 5 μl of 3 × SDS-PAGE sample buffer. Samples were resolved by 10% SDS-PAGE and analyzed by autoradiography and phosphorimaging.

### Tryptic phosphopeptide mapping of Cdc25 proteins

GST-Cdc25A, B, and C (5 μg) were phosphorylated in the presence of [γ^-32^P]-ATP by coexpressed Cdk2-Speedy/Ringo A2 or [pT160]Cdk2-cyclin A as described above. [^32^P]-Cdc25 was subjected to tryptic peptide mapping as described [50]. Briefly, samples were resolved by 10% SDS-PAGE, excised from gels, and extracted in 50 mM ammonium bicarbonate, 10% β-mercaptoethanol, and 0.2% SDS. Cdc25 was precipitated with cold trichloroacetic acid (20%) in the presence of 20 μg of bovine serum albumin as a carrier. The precipitated pellets were washed twice with ice cold acetone. Cdc25 proteins were then digested with 20 μg of sequencing grade trypsin (Promega, Cat#V5111) at 37°C overnight. The tryptic peptides were separated on thin-layer cellulose plates (EM chemicals) by horizontal electrophoresis at 1,000 V for 25 min in pH 1.9 buffer (2.5% [vol/vol] formic acid and 7.8% [vol/vol] acetic acid) followed by ascending thin-layer chromatography in 32.5% [vol/vol] *n*-butanol, 25% [vol/vol] pyridine, 7.5% [vol/vol] acetic acid. Following chromatography, the plate was dried and autoradiographed.

### Phosphorylation/dephosphorylation of Cdk2-Speedy/Ringo A2

1 μg of GST-Cdk2^D145N ^was preincubated with 4 μg of GST-Speedy/Ringo A2 in 10 μl of 1 × EB at room temperature for 20 min. Phosphorylation of Cdk2 on the activating phosphorylation site by yeast Cak1p and mammalian CAK was carried out as described [38]. The reaction was terminated by the addition of 3 × SDS-PAGE sample buffer. Samples were resolved in 10% SDS-PAGE and analyzed by autoradiography.

^32^P-labeled GST-Cdk2^D145N ^was prepared and purified as described previously [39]. The dephosphorylation of ^32^P-Cdk2 by PP2C were carried out as described [39,40]. Briefly, ~80 ng of ^32^P-Cdk2 was incubated with an excess of cyclin A (0.5 μg), GST-Speedy/Ringo A2 (1 μg), or buffer A at room temperature for 20 min. Samples were incubated at room temperature for 10 min following the addition of 100 ng of recombinant human PP2Cα [40]. Samples were analyzed by 10% SDS-PAGE as described above.

## List of abbreviations

CAK, Cdk-Activating Kinase; CDK, cyclin-dependent protein kinase; Spy1, Speedy; GST, glutathione *S*-transferase; PAGE, polyacrylamide gel electrophoresis.

## Authors' contributions

SG carried out the experiments described in Figure 3A and performed preliminary versions of most of the experiments shown in Figures 2 and 3. PK carried out the experiment described in Figure 7B. AC carried out all other experiments. AC drafted the manuscript and prepared the figures. MS conceived the project, participated in its design and execution, and revised the manuscript. All authors participated in revising the manuscript and have read and approved the final manuscript.
